# Picosecond electrical response in graphene/MoTe_2_ heterojunction with high responsivity in the near infrared region

**DOI:** 10.1016/j.fmre.2021.09.018

**Published:** 2021-11-09

**Authors:** Zhouxiaosong Zeng, Kai Braun, Cuihuan Ge, Martin Eberle, Chenguang Zhu, Xingxia Sun, Xin Yang, Jiali Yi, Delang Liang, Yufan Wang, Lanyu Huang, Ziyu Luo, Dong Li, Anlian Pan, Xiao Wang

**Affiliations:** a School of Physics and Electronics, Hunan University, Changsha 410082, China; b Institute of Physical and Theoretical Chemistry and LISA+, University of Tübingen, Auf der Morgenstelle 18, Tübingen 72076, Germany; c Key Laboratory for Micro-Nano Physics and Technology of Hunan Province, College of Materials Science and Engineering, Hunan University, Changsha 410082, China

**Keywords:** Two-dimension material, Graphene/MoTe_2_ heterojunction, Near infrared photodetector, Scanning photocurrent microscopy, Time-resolved photocurrent

## Abstract

Understanding the fundamental charge carrier dynamics is of great significance for photodetectors with both high speed and high responsivity. Devices based on two-dimensional (2D) transition metal dichalcogenides can exhibit picosecond photoresponse speed. However, 2D materials naturally have low absorption, and when increasing thickness to gain higher responsivity, the response time usually slows to nanoseconds, limiting their photodetection performance. Here, by taking time-resolved photocurrent measurements, we demonstrated that graphene/MoTe_2_ van der Waals heterojunctions realize a fast 10 ps photoresponse time owing to the reduced average photocurrent drift time in the heterojunction, which is fundamentally distinct from traditional Dirac semimetal photodetectors such as graphene or Cd_3_As_2_ and implies a photodetection bandwidth as wide as 100 GHz. Furthermore, we found that an additional charge carrier transport channel provided by graphene can effectively decrease the photocurrent recombination loss to the entire device, preserving a high responsivity in the near-infrared region. Our study provides a deeper understanding of the ultrafast electrical response in van der Waals heterojunctions and offers a promising approach for the realization of photodetectors with both high responsivity and ultrafast electrical response.

## 1 Introduction

Understanding the ultrafast charge carrier dynamics in various electronic and optoelectronic devices, especially in photodetectors with high response speed and efficient detection responsivity [1], [2], [3], is indispensable for enhancing their performance. Two-dimensional (2D) layered materials hold great promise for the next generation of high-efficiency photodetectors, owing to their ultrathin thickness, broadband photoresponse and high on/off ratio [4], [5], [6], [7], [8], [9]. When these 2D materials are thin, their intrinsic response speed is only approximately several picoseconds because of their atomic thickness [10,11]. However, to enhance the light-matter interaction for a larger photo-generated carrier concentration, an increase in thickness is required. Unfortunately, this could extend the intrinsic response time of the materials to the nanosecond time scale [12,13], and lead to a photodetector with high responsivity but slow response speed. Therefore, it is desirable to realize 2D layered photodetectors that possess high responsivity while maintaining an intrinsic response speed in the order of picoseconds.

One alternative approach is to construct 2D van der Waals heterojunctions that not only combine the merits of a single material but also lead to novel optical and electronic properties [14,15]. Regarding the dynamics of photocurrent, several processes contribute to the response time, including photocarrier recombination (*τ*_r_), photocarrier drift (*τ*_d_), exciton dissociation (*τ*_s_) and charge transfer (*τ*_s_) [12]. Among these processes, charge transfer (CT), which serves as an interlayer interaction in heterojunctions, involves a series of transient carrier dynamic processes [16], [17], [18], [19]. CT processes have been intensively studied using ultrafast all-optical pump-probe measurements [20], [21], [22]. Typically, a faster carrier decay in a strongly coupled graphene/WS_2_ heterojunction compared to a pure WS_2_ flake is observed, and direct CT absorption from graphene to a WS_2_ flake is explained under excitation below the WS_2_ bandgap [22]. These dynamic processes in heterojunctions indicate that interfacial CT can effectively decrease charge carrier decay time and is significant for photovoltaic and photodetector device applications. Although pure optical measurements provide information on the photo-generated carrier dynamics, a complete picture of the photoresponse involving photocurrent generation and collection in a practical device is still elusive. Recently, ultrafast time-resolved photocurrent (TRPC) measurements have been demonstrated to enable the achievement of a picosecond intrinsic response time for different 2D materials and their heterojunction-based photodetectors [10,11,23]. For instance, in graphene-covered WSe_2_ heterojunction photodetectors, a 5.5 ps response speed was realized, but with the increase of the WSe_2_ flake thickness from monolayer to 40 nm; an extended response time of 10 ns was also observed [12]. Meanwhile, using the same measurement technique, another study suggested that there were two different response speeds for the respective materials in graphene/1L MoS_2_ heterojunctions [24]. Although the drift process (*τ*_d_) of charge carriers in photocurrent generation has been studied, the contribution of the CT (*τ*_s_) process to the response speed has not been clarified.

In this work, we demonstrated a comprehensive understanding of interlayer interactions and qualitatively explained the photogenerated charge carrier transfer behaviors in graphene/MoTe_2_ van der Waals heterojunction photodetectors. Using a TRPC setup, the ultrafast response speed in pure MoTe_2_ and graphene/MoTe_2_ heterojunctions was investigated. Our results show a significantly reduced response time of MoTe_2_ in graphene/MoTe_2_ devices, which can be scaled down to 10 ps by an additional decay component introduced by the formation of the heterojunction, while the intrinsic response time of pure thick MoTe_2_ is approximately 1 ns. The heterojunction devices take full advantage of the fast response speed in graphene as well as high responsivity in MoTe_2_, and present a possible method to enhance photodetection performance by decreasing photocurrent recombination loss.

## 2 Methods

Device fabrication: Pure MoTe_2_ devices and graphene/MoTe_2_ devices were fabricated from mechanically exfoliated materials. In the pure MoTe_2_ device, a MoTe_2_ flake was mechanically exfoliated onto transparent polydimethylsiloxane (PDMS), which was then transferred onto a silicon substrate with a 300 nm-thick silicon dioxide layer. In the graphene/MoTe_2_ heterojunction devices, graphene flakes were first mechanically exfoliated onto a silicon/silicon dioxide substrate. The MoTe_2_ flake was placed on the PDMS using a similar method, and it was then aligned and transferred onto the graphene using a microscope. Cr/Au (10 nm/50 nm) conducting electrodes on top of 2D MoTe_2_ or graphene/MoTe_2_ with a channel length of 5 μm were fabricated using standard electron beam lithography (EBL), metal thermal evaporation and lift-off processes.

Basic characterization: Atomic force microscopy (AFM) (Bruker Dimension Icon) in the taping mode was used to identify the thickness of the samples. Raman measurements of the samples were taken using a confocal microscope (WITec, alpha-300) equipped with a 100× objective lens. The excitation source of the Raman spectra was a 532 nm continuous wave laser, and the laser beam was focused to 1 μm on the devices. The electrical properties were measured with an Agilent-B1500 semiconductor analyzer in a LakeShore vacuum chamber of 10^−4^ Pa.

SPCM and TRPC measurements: Scanning photocurrent microscopy (SPCM) and time-resolved photocurrent measurements (TRPC) were performed on our home-built setup. In SPCM measurements, a 780 nm fiber laser (NPI Rainbow 780 OEM) with a pulse width of 80 fs and a 488 nm continuous wave laser were chopped by a mechanical chopper at 1050 Hz, and then focused onto the sample by a long working distance objective (Olympus LMPLFLN 50×) near the diffraction limit. The generated photocurrent was collected by a lock-in amplifier (Stanford SR830) at the chopped frequency with a background noise of around 0.2 pA. The SPCM measurements were performed by raster scanning the entire device mounted on a piezoelectric translation stage (Piezoconcept LT3) according to the fixed laser spot. In TRPC studies, a 780 nm pulse laser was split into two independent beams to form a pump-probe measurement configuration. The pump beam was delayed by different path lengths, with the delay time precisely controlled by a mechanical delay stage (Thorlabs DDSM100/M). The pump and probe beams were recombined by a beam splitter after the delay line stage, and focused onto the sample using the same long working distance objective.

## 3 TRPC measurement technique and CT hypothesis

**Fig. 1:**
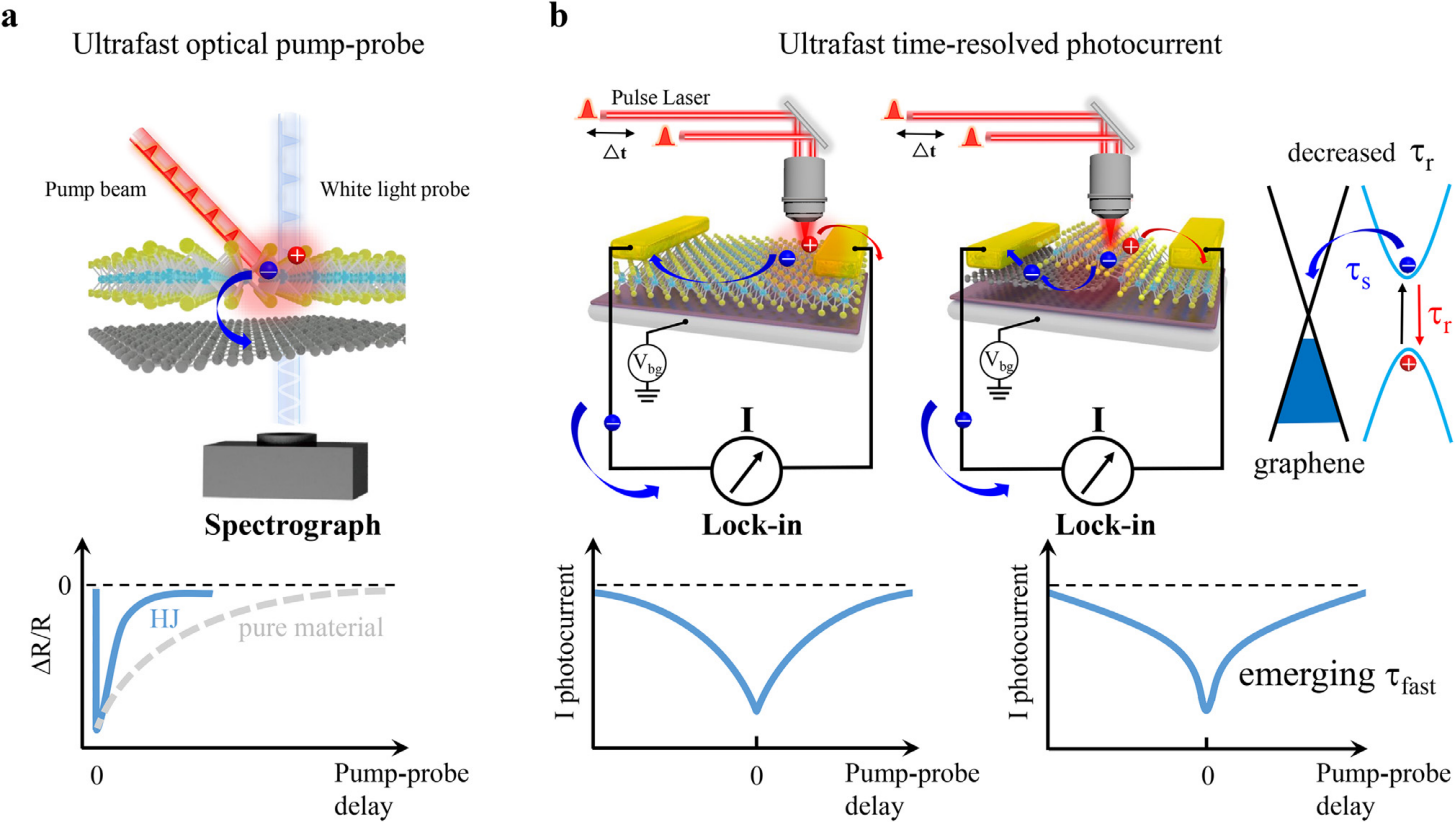
**Comparison of the ultrafast all-optical pump probe and ultrafast time-resolved photocurrent measurement.** (a) Schematic illustration of the ultrafast pure optical pump-probe measurement. The heterojunctions (light blue) show a faster carrier decay than pure materials (grey) in the transient dynamic spectrum; (b) Schematic illustration of the time-resolved photocurrent (TRPC) measurement. Left: Photocurrent generation in pure 2D TMDCs by the photovoltaic effect, where the recovery of saturated photocurrent corresponds to a single component photocurrent response in the TRPC spectrum. Right: Photocurrent generation in Gr/TMDCs heterojunction by the photovoltaic effect, where the new charge carrier's transfer channel provided by graphene effectively decreases the recombination loss and brings about a fast photocurrent response component in the TRPC spectrum.

Ultrafast carrier dynamics in materials can be probed by the all-optical pump-probe technique, as shown in the transient absorption measurements (Fig. 1a). In these measurements, a pulsed pump laser beam with a specific energy is typically used to selectively excite the sample, and another pulsed white laser serving as a probe beam can be applied to trace the desired signals, such as the interfacial CT process. The time-resolved differential transmission spectrum can be expressed as △*T*/*T*_0_ = (*T* - *T*_0_)/*T*_0_, where *T* and *T*_0_ are the white light-induced transmission signals with and without the pump beam, respectively. Hence, positive signals represent absorption saturation and photo-induced bleaching in the ground state. With different time delays between the pump and probe pulse, the differential transmission collected by the spectrograph indicates the carrier dynamic changes. In pure 2D materials, the differential transmission generally exhibits a slow exponential decay with an increase in the delay time, corresponding to the exciton formation process and its recombination lifetime [25] (Fig. 1a lower plane, gray curve). In the construction of heterojunctions, an enhanced signal intensity and an exponential decay with a much faster process than pure materials can be observed, demonstrating a CT process to the adjacent material (Fig. 1a lower plane, light blue curve). In addition, with specific probe energy (usually below the bandgap of the charge carrier extraction material), the rise time in the differential transmission spectrum of the carrier injection material can further reflect the CT time scale [26]. Although all these optical measurements provide important information on the intrinsic photogenerated carrier dynamics, which can be considered the ultimate limit of the photoresponse in the materials, these processes may not contribute to the photocurrent or other electric responses in a practical device.

Compared to the all-optical pump-probe experiments, the TRPC measurements follow the conventional pump-probe scheme, where a pulsed laser beam (80 fs in this work) is split into two independent beams to form a pump-probe configuration, and an ultrafast photo-generated current can be detected with a sub-picosecond resolution (Fig. S1). Crucially, the pump in the TRPC measurements must be sufficiently strong to saturate the sample. When the pump beam and the probe beam coincide spatially and temporally, the generation of photocurrent by the probe beam is suppressed because of the saturation in the ground state, causing a prominent dip to appear at the zero-time delay (Fig. 1b lower plane). With an increase in the delay time between the pump and probe beam, parts of the pump-induced charge carriers relax, and can be excited again by the probe beam. Accordingly, the time-resolved photocurrent displays an exponential decay (recovery) with *τ* corresponding to the device response time. In all the processes contributing to the response time, photocarrier recombination (*τ*_r_) leads to photocurrent loss, whereas photocarrier drift (*τ*_d_) in combination with exciton dissociation and CT (*τ*_s_) leads to the generation of photocurrent. Meanwhile, the response time can be expressed using the equation *τ*^−1^ = (*τ*_d_ + *τ*_s_) ^-1^ + *τ*_r_^−1^ [12]. Hence, the TRPC generation in pure 2D materials follows a single-exponential decay with only CT to the electrodes (Fig. 1b left). In contrast, according to the studies on all-optical pump probes, when forming a heterojunction, such as graphene and MoTe_2_ flakes (Fig. 1b right), the graphene layer provides an additional CT channel, and the band alignment of the straddling gap (type I) can lead to photo-excited charge carriers in MoTe_2_ transferring to the graphene (*τ*_s_). With less exciton recombination in the light-absorption material, the photocurrent recombination loss (*τ*_r_) of the entire device is reduced, and a new fast decay component is expected to emerge in the TRPC measurement (Fig. 1b right).

## 4 Comparison of photodetection performance in pure MoTe_2_ and Gr/MoTe_2_ devices

**Fig. 2:**
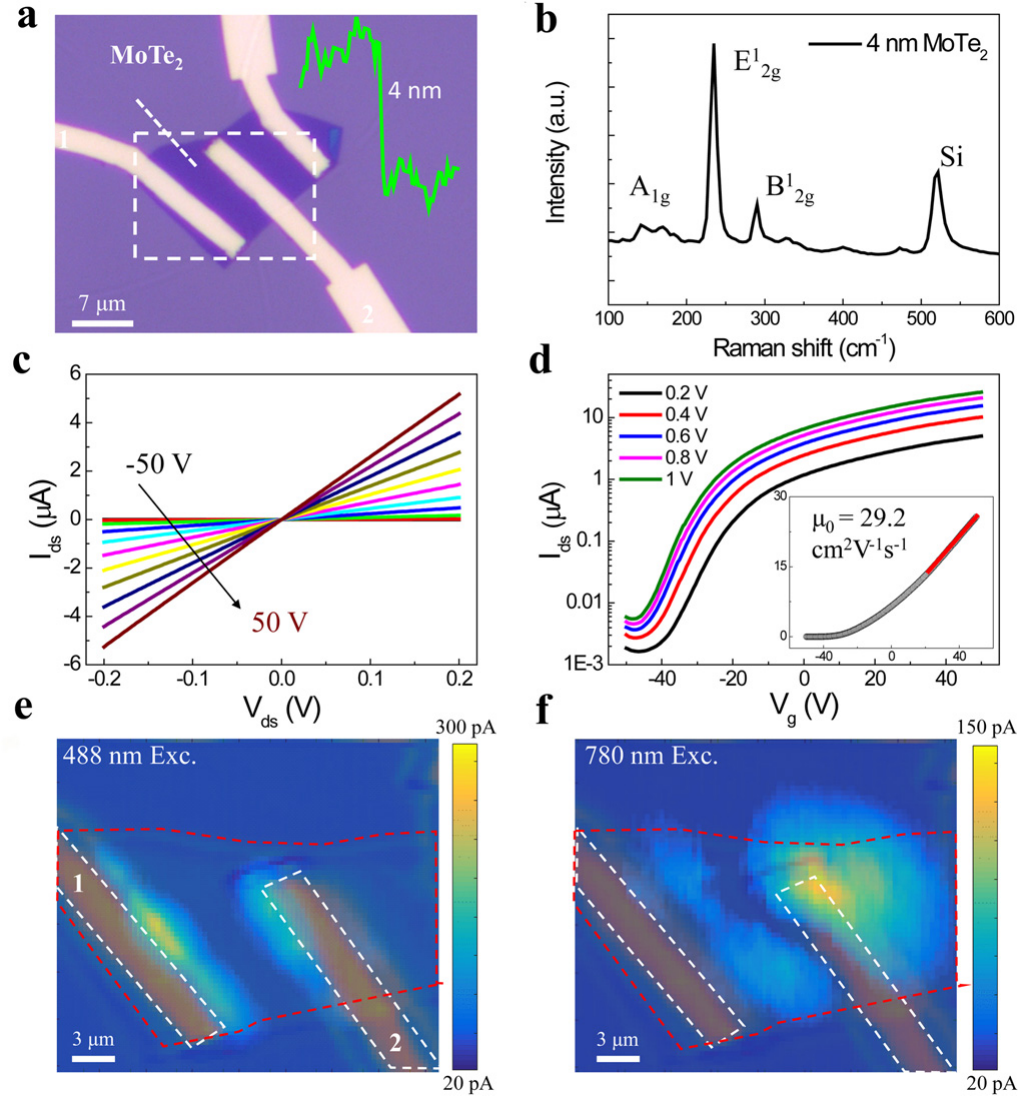
**Electrical transport characterizations and photoresponse in the 4 nm pure MoTe**_**2**_**device.** (a) Optical image of the fabricated MoTe_2_ device. Inset: AFM line scan performed along the white dashed line shown in the optical image; (b) corresponding Raman spectrum, where the three prominent peaks A_1g_, E^1^_2g_ and B^1^_2g_ are identified to be the fingerprint of the thin layered MoTe_2_; (c) *I*_ds_-*V*_ds_ output characteristic curve at gate voltages varying from -50 V to 50 V; (d) *I*_ds_-*V*_g_ transfer characteristic curve at source-drain voltage from 0.2 to 1 V on the semi-logarithmic scale. Inset shows the mobility of this device around to be 29.2 cm^2^ V^−1^s^−1^; (e) and (f) Scanning photocurrent maps (SPCM) of the MoTe_2_ device at 0 V source-drain voltage excited by a 488 nm CW laser and 780 nm pulse laser, respectively.

To elucidate the distinct charge carrier dynamic processes, we first investigated the ultrafast photocurrent behaviors in photodetectors made of pure MoTe_2_ layers (see Methods). Fig. 2a shows an optical image of a pure MoTe_2_ photodetector with a thickness of 4 nm, and the corresponding Raman spectrum (Fig. 2b) exhibiting three characteristic peaks located at ∼171, ∼234, and ∼289 cm^−1^ were assigned to the A_1g_, E^1^_2g_ and B^1^_2g_ vibration modes, respectively [27]. In the electrical transport characteristics, the *I*_ds_-*V*_ds_ output characteristic curve at room temperature in this device shows a linear current change at the back-gate varying from -50 V to 50 V (Fig. 2c), indicating a small contact barrier between MoTe_2_ and the metal electrodes. With the increase in V_g_, the conductance of the MoTe_2_ device is monotonically enhanced, demonstrating an n-type transport behavior. Meanwhile, in the *I*_ds_-*V*_g_ transfer characteristic curve, this device exhibits an on/off ratio of approximately 10^4^ with a threshold voltage of -40 V, manifesting an electron-doped intrinsic property (Fig. 2d). Based on the transconductance, we calculate the electron mobility of this two-terminal device using the equation *μ*_0_ = [d*I*_ds_/d*V*_bg_] × [*L*/*WC*_i_*V*_ds_] [28], where *L*/*W* is the ratio between the channel length and width, and *C*_i_ is the capacitance between the back gate per unit area (in our case *C*_i_ = 1.15 × 10^−8^ F cm^−2^ for 300 nm thick silicon dioxide). The result reveals a value around 29.2 cm^2^ V^−1^s^−1^, which is among the high mobilities in various transition metal dichalcogenides (TMDCs) [29].

The spatially resolved photocurrent response (see Method) of the MoTe_2_ device was characterized by a home-built scanning photocurrent microscopy (SPCM) at zero bias under the excitation of a continuous-wave 488 nm laser (Fig. 2e) and a pulsed 780 nm laser (Fig. 2f). To better determine the position of the generated photocurrent, the optical reflection images of the devices were superimposed on the photocurrent images in Fig. 2e, f with the white dashed rectangles indicating the locations of the electrodes. Apparently, these two SPCM images recorded at zero bias display the photocurrent appearing at the electrode edges, which is attributed to the contribution of both PV and photo-thermoelectric effect (PTE) [30], [31], [32]. While the broadened photocurrent in Fig. 2f can be ascribed to the enhanced photothermoelectric effect and reduced optical resolution by the excitation of the 780 nm pulse laser. The excitation laser power-dependent photocurrent response was also investigated for the MoTe_2_ photodetector, and similar SPCM images were obtained with an increase in laser power (Supplementary Section 2). Based on the photocurrents measured by the SPCM, we have calculated the responsivity of the 4 nm thick MoTe_2_ photodetector at zero bias and relatively low excitation powers, yielding 0.6 mA W^−1^ at 488 nm and 0.23 mA W^−1^ at 780 nm. These obtained responsivities are comparable to those of previously reported thin-layered TMDCs under a similar experimental configuration [24,33,34].

**Fig. 3:**
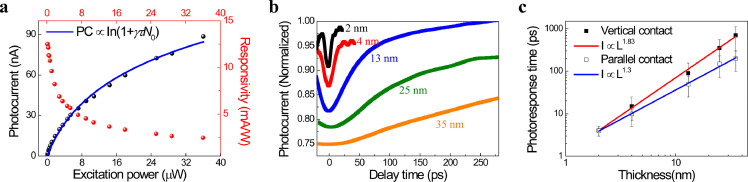
**Responsivity and photocurrent response speed in pure MoTe**_**2**_**devices with different thicknesses revealed by TRPC technique.** (a) Photocurrent (left) and responsivity (right) as the function of probe power in a 25 nm pure MoTe_2_ device. The solid blue line in probe-induced photocurrent fits the formula I_PC_ ∝ In(1 + *γτN*); (b) TRPC measurements show extended response time with the increase of the MoTe_2_ thicknesses; (c) Response time (*τ*) as the function of their thicknesses (*L*) on the log-log scale. The open squares are the *τ* of the pure two-terminal MoTe_2_ devices with the parallel contacts in Fig. (b), while the solid squares are derived from the longer *τ* component in Gr/MoTe_2_ heterojunction with vertical contacts. The red and blue solid fitting curves display the *τ* ∝ L^1.83^ and *τ* ∝ L^1.3^ in the vertical and parallel electrode configurations, respectively. The data points are the average values of the response time and the error bars indicate the minimum and the maximum values.

To gain more insight into the photocurrent detection performance of these infrared materials, we fabricated other MoTe_2_ photodetectors with the thickness *L* varying from 2 nm to 35 nm. Fig. 3a presents the photoresponse of a representative 25 nm thick photodetector using a single probe laser beam at 780 nm. We observe a typical sublinear power dependence (black spheres), which can be fitted by the equation PC ∝ ln(1 + *γτN*_0_) [13,23] (blue line), where *γ* is the Auger recombination rate, *τ* is the response time, and *N*_0_ is the initial exciton population for each pulse. We further calculated the responsivity of this 25 nm MoTe_2_ photodetector at different excitation powers, according to the experimentally obtained photocurrents. At zero source-drain bias, the highest value of 12.5 mA W^−1^ is obtained at the excitation power of 0.36 μW, almost two orders of magnitude larger than the 4 nm MoTe_2_ device and several times higher than those of other high-speed photodetectors operating in the near-infrared region [14,35,36].

The response speed of the two-terminal pure MoTe_2_ photodetectors with different thicknesses *L* was then examined by pump-probe ultrafast photocurrent measurement (see Method). Here, the probe beam at 780 nm was chopped so that the lock-in amplifier could only measure its photocurrent. When increasing the thickness *L* from 2 to 35 nm, the response time exhibits a distinct extended trend (Fig. 3b). We extract the decay *τ* with respect to thickness *L* using the equation $\frac{\text{PC}(\Delta t)}{\text{PC}(\Delta t\to \infty )}\;=\;1\;-\;\text{Aexp}(-\frac{\Delta t}{\tau })$, and the result shows a power law relationship of approximate 1.3 (blue line in Fig. 3c). These results indicate that the extended response speed derived from the transient time *τ*_tran_ is unavoidable, even in the two-terminal lateral device configuration. We attribute this phenomenon to the fact that the photogenerated charge carriers in thicker MoTe_2_ photodetectors are initially in the middle of the samples rather than at the surface [37], and therefore, they still need to experience a longer out-of-plane distance to drift to the electrodes on the top. Thus far, the thickness-dependent photoresponse properties of pure MoTe_2_ detectors have been demonstrated, which indicate that a device with one 2D material can not possess both high responsivity and fast response speed.

**Fig. 4:**
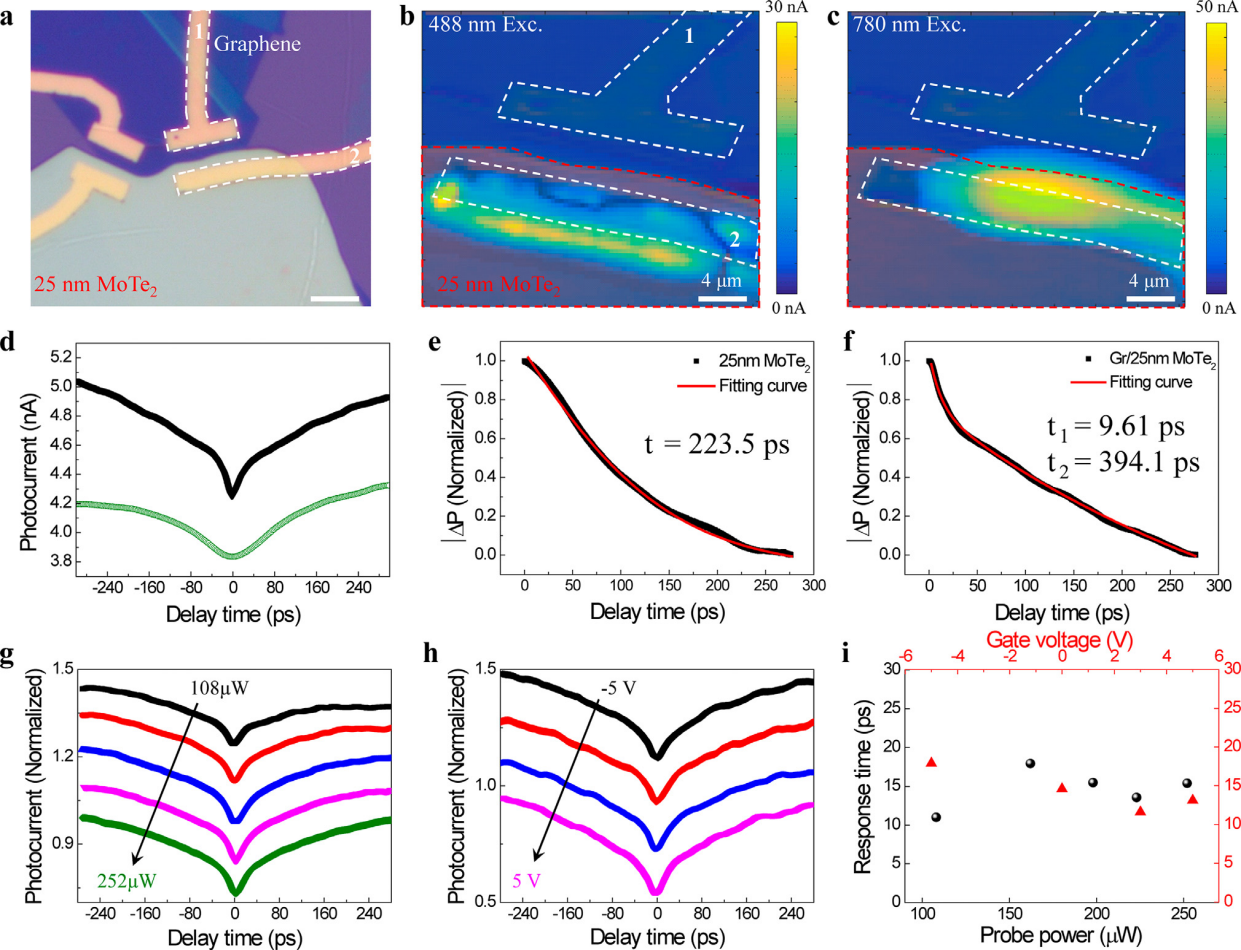
**Enhancement of photocurrent response speed by integrating of graphene with a 25 nm MoTe**_**2**_**.** (a) Optical image of the fabricated Gr/25 nm MoTe_2_ heterojunction device; (b) and (c) SPCM of the device in (a) at 0 V source-drain voltage with the excitation of a 488 nm CW laser and 780 nm pulse laser respectively. The white and red dashed curves outline the position of the electrodes and the MoTe_2_, respectively; (d) comparing TRPC in pure 25 nm MoTe_2_ (green) with the Gr/25 nm MoTe_2_ (black) heterojunction, where the heterojunction shows two prominent response time scales; (e) and (f) normalized photocurrent (|∆PC|) decay curves of pure 25 nm MoTe_2_ and the Gr/25 nm MoTe_2_ heterojunction, where the solid red lines fit the exponential decay. The |∆PC| is the difference between the photocurrent (t = 0) and photocurrent (t → ∞), with each decay curve normalized for a better comparison of different |∆PC| data; (g) Normalized TRPC in the Gr/25 nm MoTe_2_ heterojunction at probe powers from 252 μW to 108 μW; (h) normalized TRPC in Gr/25 nm MoTe_2_ heterojunction at gate voltages from -5 V to 5 V; (i) response time as a function of probe power and gate voltage in Gr/25 nm MoTe_2_ heterojunction.

Considering the ultrahigh electron mobility of graphene and the efficient CT at the heterojunction interface, we further fabricated vertical graphene/MoTe_2_ heterojunction photodetectors. Fig. 4a presents the optical image of a typical heterojunction device with a MoTe_2_ thickness of 25 nm. The corresponding SPCMs of this device obtained using different excitation lasers are shown in Fig. 4b and c. Compared with the pure 25 nm MoTe_2_ photodetector (Supplementary Section 5), the SPCMs of the graphene/MoTe_2_ heterojunction exhibited a similar photocurrent intensity and the photocurrent profile appeared only at the electrodes on top of MoTe_2_, indicating that the integration of graphene/MoTe_2_ does not form a strong band-bending at the junction (Supplementary Section 6) and does not influence the photoresponsivity of intrinsic MoTe_2_. It should be noted that the photoresponse of graphene in our heterojunction is one to two orders of magnitude weaker than that of MoTe_2_. Meanwhile, because the energy of the 780 nm pulse laser (1.59 eV) is closer to the multilayered MoTe_2_ exciton absorption (0.9 eV) [38], the heterojunction has a larger photoresponse to the red laser than the blue laser. In the TRPC experiments, we observed a distinct photocurrent decay in the MoTe_2_ region of the heterojunction. This phenomenon is also observed in the junction region where graphene and MoTe_2_ fully overlap. Compared with the pure 25 nm MoTe_2_ photodetector (green curve in Fig. 4d), a prominent fast new decay component appears at the graphene/MoTe_2_ photodetector, while the slow component is preserved (black curve in Fig. 4d). We plotted the data |∆PC| defined as |PC (*t*=0) – PC (*t*=∞)| with respect to the delay time, and used the equation $\frac{\text{PC}(\Delta t)}{\text{PC}(\Delta t\to \infty )}\;=\;1\;-\;\text{Aexp}(-\frac{\Delta t}{\tau })$ and $\frac{\text{PC}(\Delta t)}{\text{PC}(\Delta t\to \infty )}\;=\;1\;-\;\text{Aexp}(-\frac{\Delta t}{\tau 1})\;-\;\text{Bexp}(-\frac{\Delta t}{\tau 2})$ to fit the different photocurrent decay curves in pure 25 nm MoTe_2_ (Fig. 4e) and the heterojunction (Fig. 4f), respectively. For a better comparison of the different |∆PC| data, each decay curve was normalized. The decay curve in the graphene/MoTe_2_ heterojunction yields a new decay component of approximately 10 ps with a relative weight percentage of 15.5% (Fig. 4f), which is one order of magnitude faster than the intrinsic response time of ∼400 ps in 25 nm MoTe_2_ and implies a photodetection bandwidth as wide as 100 GHz. We further measured other series of heterojunction photodetectors with different MoTe_2_ thicknesses, with almost every device having this fast decay component. In the thicker (35 nm) samples, the fast response time slightly increased to 16 ps, and the relative weight percentage decreased to 10% (Supplementary Section 8). Meanwhile, in these vertical heterojunction devices, we observed a longer intrinsic MoTe_2_ response time (the slow component) than the two-terminal devices with the same thickness, which shows a power law relationship of 1.8 between *τ* and *L* (red line in Fig. 3c), corresponding to the transient time expression of *τ*_tran_ = *L*^2^/*µV*_bias_ [12,24]. To further study this fast decay component, we performed probe power and gate-dependent TRPC measurements in a graphene/25 nm MoTe_2_ heterojunction photodetector. Fig. 4g shows the normalized TRPC spectrum for different probe powers at gate bias of 0 V. With the increase in probe power from 108 μW (19 μJ cm^−2^) to 252 μW (45 μJ cm^−2^), two timescales appear at all TRPC curves and there is only a slightly enhanced dip at zero time delay. We also plotted the time-resolved |∆PC| for different probe powers and put them in one graph. Most prominently, all the normalized TRPC curves seem to overlap and show an almost unchanged fast decay component (Supplementary Fig. S7). The fitted fast response time and |∆PC| for all the probe powers are shown in Fig. 3i. The results reveal that the response time in graphene/25 nm MoTe_2_ remains at 10-15 ps independent of the probe power (black dots in Fig. 3i). In the gate-dependent TRPC measurement, we changed the back gate voltage from -5 V to 5 V. With the different gate biases, a similar result was obtained (red triangles in Fig. 4i), where the fast response time did not show any significant changes. The preserved fast decay component under different power and gate conditions indicates that the high-speed graphene/MoTe_2_ heterojunction photodetector can perform under various circumstances.

**Fig. 5:**
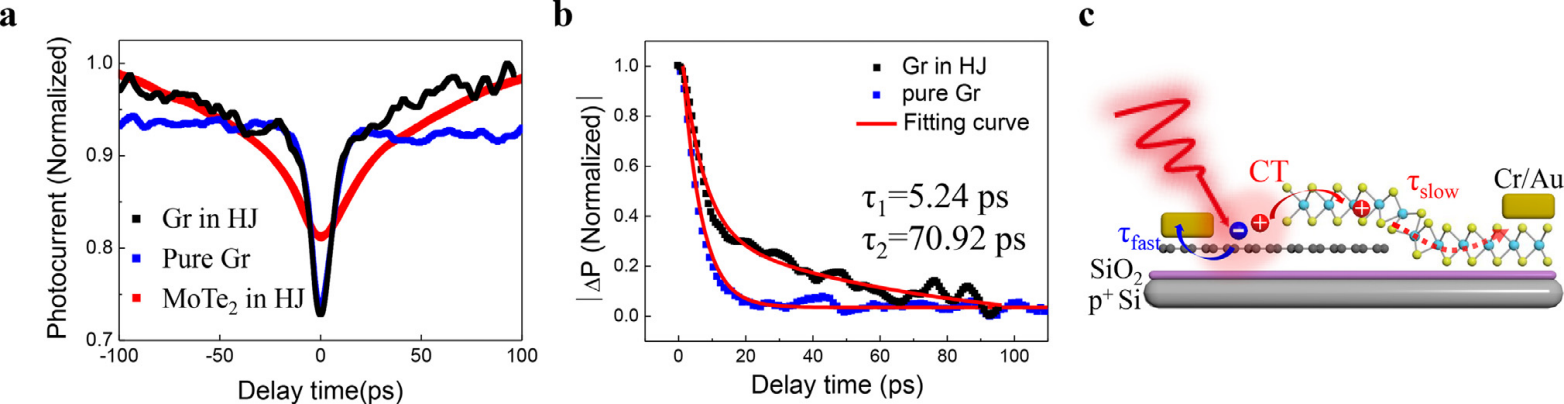
**Demonstration of charge carrier transfer in the Gr/25 nm MoTe**_**2**_**heterojunction device.**(a) TRPC of graphene (black) in Gr/MoTe_2_ heterojunction, 25 nm MoTe_2_ (red) in Gr/MoTe_2_ heterojunction and pure graphene (blue); (b) normalized photocurrent (|∆PC|) decay in pure graphene (blue squares) and graphene in heterojunction (black squares), where the two time constants in graphene of heterojunction correspond to *τ*_1_ = 5.24 ps and *τ*_2_ = 70.92 ps; (c) schematic illustration of the photo-induced charge carrier transfer and two different response components in Gr/MoTe_2_ heterojunction when the graphene region is excited. The dashed red arrow indicates the slower response component due to a relatively low mobility in thick MoTe_2_.

To prove the origin of the fast photocurrent decay component, we further conducted TRPC measurements in the graphene region of the heterojunction. In contrast to the pure graphene (blue curve in Fig. 5a) or the MoTe_2_ region in the heterojunction (red curve in Fig. 5a), a photocurrent decay comparable to that of intrinsic graphene (∼5 ps, 63.5% of weight) with a slower component (∼70 ps, 36.5% of weight) is observed in the graphene region of the heterojunction. According to previous reports, the photogenerated electron-hole pairs in some unique Dirac semimetals such as graphene [11,39,40] or Cd_3_As_2_ [35] can relax their absorbed energy through rapid electron-electron interactions without any change in the lattice temperature. Hence, intrinsic graphene possesses an ultrafast photocurrent response of several picoseconds [41], independent of the pump laser with an increase in time delay (blue square in Fig. 5b). Accordingly, the observed slow decay component is attributed to the interaction between graphene and MoTe_2_ (Fig. 5c), which also gives rise to the fast photocurrent decay in MoTe_2_ of the graphene/MoTe_2_ heterojunction photodetector.

## 5 Mechanism discussion

The observed different photo-response speeds with an extra component in the respective materials at heterojunctions suggest a possible method for the realization of photodetectors with high response speed and efficient detection responsivity. We used the photocurrent generation model to further interpret and discuss the ultrafast response time and the dynamic process of charge carriers in graphene/MoTe_2_ heterojunction photodetectors. As proposed at the beginning, the response time in the generation of photocurrent can be expressed using the equation *τ* ^−1^ = (*τ*_d_ + *τ*_s_) ^-1^ + *τ*_r_^−1^. To analyze the complicated dynamics in the heterojunction, we first consider pure graphene and pure 25 nm MoTe_2_. For pure graphene, the average response time *τ* is approximately several picoseconds for the multilayer used in this study. In our case, we take the maximum response time as the photocarrier recombination time *τ*_r_ and ignore *τ*_s_, because the exciton dissociation time *τ*_s_ is far smaller than the response time. It follows that the photocarrier drift time *τ*_d_ in pure graphene is equal to $\tau_{r}\;\cdot \;\tau /\left(\tau_{r\;}-\;\tau \right)\;=$ 8 ps (here, we take 3.75 ps for *τ* and 7 ps for *τ*_r_). Likewise, a drift time *τ*_d_ of approximatedly 2000 ps was obtained for 25 nm pure MoTe_2_. The significantly shorter drift time *τ*_d_ obtained in pure graphene compared to that found in MoTe_2_ is consistent with the fact that graphene has a significantly larger in-plane and out-of-plane mobility than MoTe_2_.

Next, we discuss the situation in the graphene/MoTe_2_ heterojunction, where we observed two different response times in the respective materials. We believe that there are two possible mechanisms for the recombination of charge carriers in this heterojunction. One possibility is that the recombination time in the heterojunction is the average time between graphene and MoTe_2_, and the excited charge carriers of different materials in heterojunction have the same recombination process. In this case, the slower intrinsic response time component in thick MoTe_2_ requires a recombination time longer than 400 ps (*τ*_r MoTe2_^−1^ = *τ*_MoTe2_^−1^ - (*τ*_d MoTe2_ + *τ*_s MoTe2_) ^-1^ < *τ*_MoTe2_^−1^, and *τ*_r MoTe2_ > *τ*_MoTe2_ = 400 ps). With identical recombination times, the photocarrier drift time, exciton dissociation time, and charge transfer time (*τ*_d_ + *τ*_s_) in graphene are equal to (*τ*_graphene_^−1^ - *τ*_r graphene(MoTe2)_^−1^)^−1^ < (*τ*_graphene_^−1^ - 400^−1^)^−1^, which is shorter than that of pure graphene, leading to a contradiction of the physical facts.

After excluding the process occurring with an average time, we propose that in the graphene/MoTe_2_ heterojunction, graphene and MoTe_2_ follow their own recombination time. In this case, if we consider the fast photocurrent decay component in MoTe_2_ of the graphene/MoTe_2_ heterojunction, then a drift time far smaller than the intrinsic MoTe_2_ can be obtained (*τ*_MoTe2_^−1^ = (*τ*_d HJ_ + *τ*_s HJ_)^−1^ + *τ*_r MoTe2_^−1^. Here we take 10 ps for *τ*_MoTe2_ and 20 ps for *τ*_r MoTe2_). If we further use a quick charge transfer time of 5 ps between MoTe_2_ and graphene [25], a drift time of 15 ps is obtained in the heterojunction, which is two orders of magnitude smaller than the thick intrinsic MoTe_2_. Therefore, we can conclude that the fast response time of the CT process in the graphene/MoTe_2_ heterojunction decreases the photocurrent recombination loss in thick MoTe_2_, and the graphene leads these parts of the transferred carriers to have a shortened drift time. In principle, this structure makes full use of the ultrahigh mobility in graphene and breaks the limitation of transient time.

## 6 Conclusion

In summary, using time-resolved photocurrent measurements, we investigated the response time in pure MoTe_2_ and graphene/MoTe_2_ heterojunction photodetectors with different thicknesses. We demonstrated that the extended response time in pure thick MoTe_2_ can be scaled down to 10 ps with the integration of graphene. By doing so, we realized a high-performance photodetector with both high responsivity and quick response by making use of the CT process and large carrier mobility in graphene. The design of an ultrafast device and the understanding of carrier dynamics in graphene/MoTe_2_ heterojunction photodetectors pave the way for the next generation of ultrathin van der Waals optoelectronic devices.

## Declaration of Competing Interest

The authors declare that they have no conflicts of interest in this work.

## Appendix Supplementary materials

Image, application 1
