# Supplementary material for: Picosecond electrical response in graphene/MoTe2 heterojunction with high responsivity in the near infrared region
Source: Fundam Res. 2021 Nov 9;2(3):405–11. doi: 10.1016/j.fmre.2021.09.018 (PMC11197620; doi:10.1016/j.fmre.2021.09.018)
Supplement: Supplementary file 1 [file mmc1.docx]

**Supplementary Information**

**Picosecond electrical response in Gr/MoTe_2_ heterojunction with high responsivity in the near infrared region**

Zhouxiaosong Zeng^a,1^, Kai Braun^b,1^, Cuihuan Ge^a,1^, Martin Eberle^b^, Chenguang Zhu^c^, Xingxia Sun^c^, Xin Yang^c^, Jiali Yi^c^, Delang Liang^c^, Yufan Wang^a^, Lanyu Huang^a^, Ziyu Luo^c^, Dong Li^c^, Anlian Pan^c^*, Xiao Wang^a^*

*^a^School of Physics and Electronics, Hunan University, Changsha 410082, China*

*^b^Institute of Physical and Theoretical Chemistry and LISA+, University of Tübingen, Auf der Morgenstelle 18, 72076, Tübingen, Germany*

*^c^Key Laboratory for Micro-Nano Physics and Technology of Hunan Province, College of Materials Science and Engineering, Hunan University, Changsha, 410082, China*

*^1^ These authors contributed equally to this work.*

*Corresponding authors:

*E-mail* addresses: [anlian.pan@hnu.edu.cn](mailto:anlian.pan@hnu.edu.cn)(A.L. Pan), [xiao_wang@hnu.edu.cn(X](mailto:xiao_wang@hnu.edu.cn(X). Wang)

This supplementary information contains a 11-page document, including 10 figures and this cover page.

1. **Time-resolve photocurrent set-up.**

Fig. S1a shows the optical elements and optical path of our home-built time-resolved photocurrent (TRPC) set-up. Fig. S1b is the schematic illustration of the development of the free carrier distribution induced by the probe beam. When the time delay between the pump (red) and probe (orange) beam is smaller than the carrier lifetime (upper panel in Fig. S1b), the pump beam saturates the ground state, and the free carriers excited by the probe beam are reduced. For a large delay time between the pump and probe beams, a large portion of the free carriers relax to the ground state and can be re-excited by the probe beam (lower panel in Fig. S1b). Hence, along with the increase of the delay time, the photocurrent recorded by the lock-in amplifier recovers gradually.


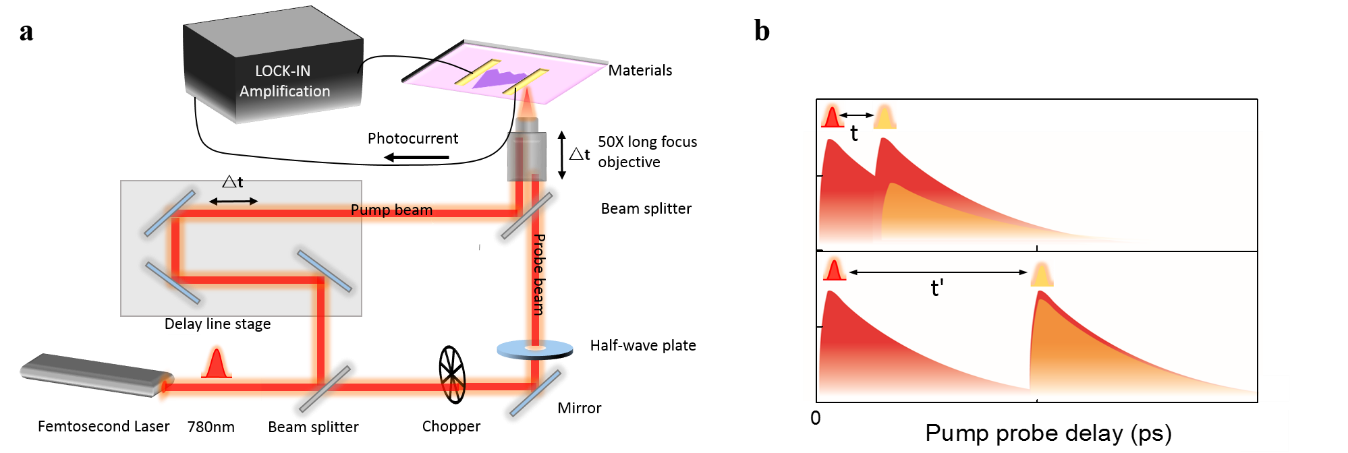


**Fig. S1** (a) Experimental set-up for the time-resolved photocurrent measurement; (b) Schematic illustration of the development of free carrier distribution induced by the probe beam at different pump-probe delays.

1. **Power dependent scanning photocurrent microscopy measurements.**

We perform power dependent scanning photocurrent microscopy (SPCM) measurements on the 4 nm thick pure MoTe_2_ device by varying the excitation laser powers under both the 488 nm continuous wave (CW) laser and 780 nm pulse laser excitations. We observe all intense photocurrent patterns appearing at the edges of electrodes, which are similar to the phenomenon shown in the main text.


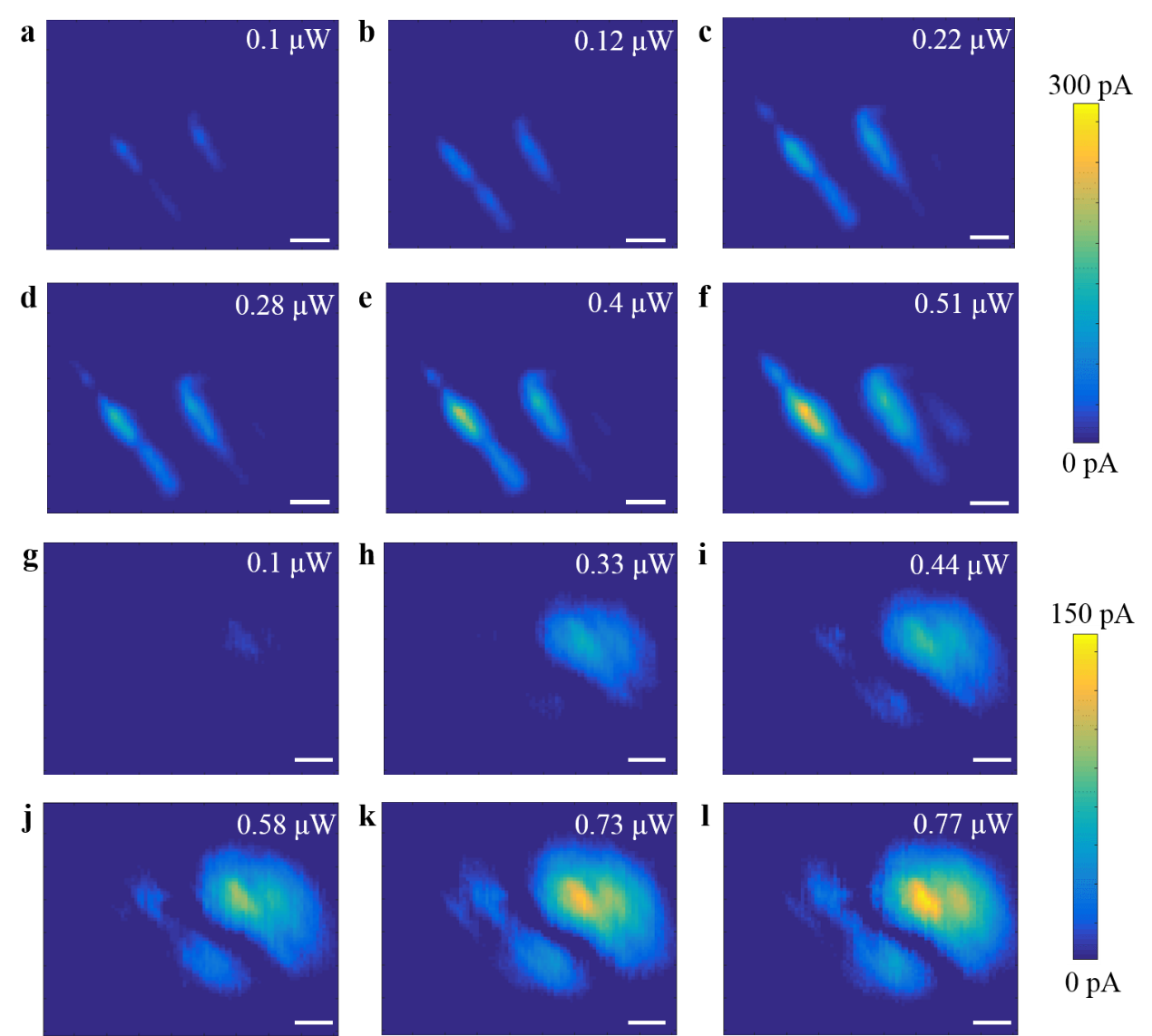


**Fig. S2** (a-f) Power dependent SPCM images excited by the 488 nm CW laser; (g-l) Power dependent SPCM images excited by the 780 nm pulse laser. All the scale bars are 2.5 μm.

1. **Atomic force microscopy characterizations of MoTe_2_ samples.**

Atomic force microscopy (AFM) in taping mode was used to identify the thickness of the exfoliated MoTe_2_ samples on the silicon/silicon dioxide substrate. The MoTe_2_ samples shown in Fig. S3 (a-f) corresponds to the photodetectors in the main text with different thicknesses.


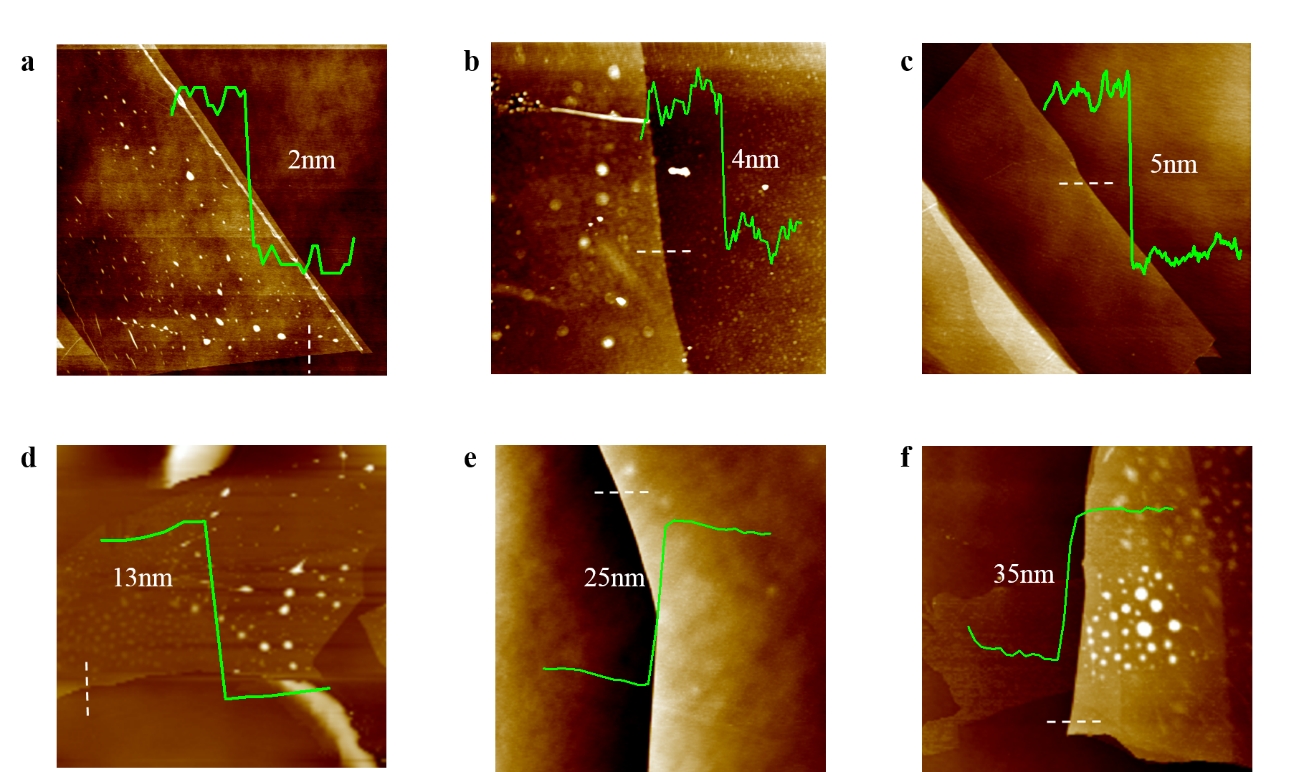


**Fig. S3** (a-f) AFM characterizations of MoTe_2_ samples with different thicknesses.

1. **Raman spectra of graphene/MoTe_2_ device.**

Raman spectroscopy were performed to characterize the graphene/MoTe_2_ heterojunctions. As shown in Fig. S4, typical Raman signatures of graphene (G peak at 1582 cm^−1^ and 2D peak at 2700 cm^−1^) , and MoTe_2_ (A_1g_ peak at 171 cm^−1^ , E^1^_2g_ peak at 234 cm^−1^ and B^1^_2g_ peak at 289 cm^−1^)[[1](#_ENREF_1)] were observed.


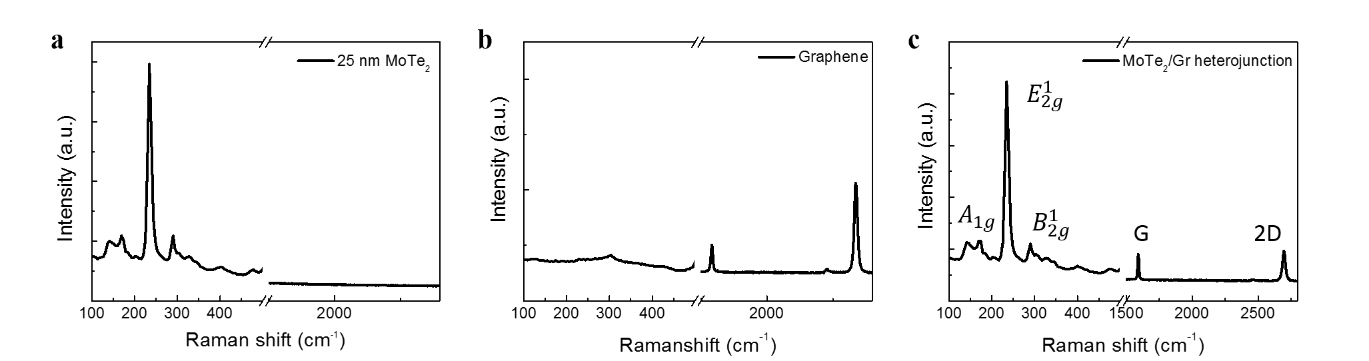


**Fig. S4** Raman spectra of (a) pure 25 nm MoTe_2_; (b) pure graphene, and (c) Gr/MoTe_2_ heterojunction.

1. **SPCM of pure 25 nm MoTe_2_.**

We also performed SPCM of a 25 nm thick pure MoTe_2_ photodetector to compare with the graphene/25 nm MoTe_2_ photodetector discussed in the main text. Fig. S5 a and b are the low and high magnification optical images of the 25 nm pure MoTe_2_ photodetector, which shares the same MoTe_2_ flake with the graphene/MoTe_2_ photodetector. In the SPCM images (Fig. S5 c and d), prominent photocurrent intensity patterns appear at the center between two electrodes, which is attributed to the large carrier diffusion length in the thick sample.


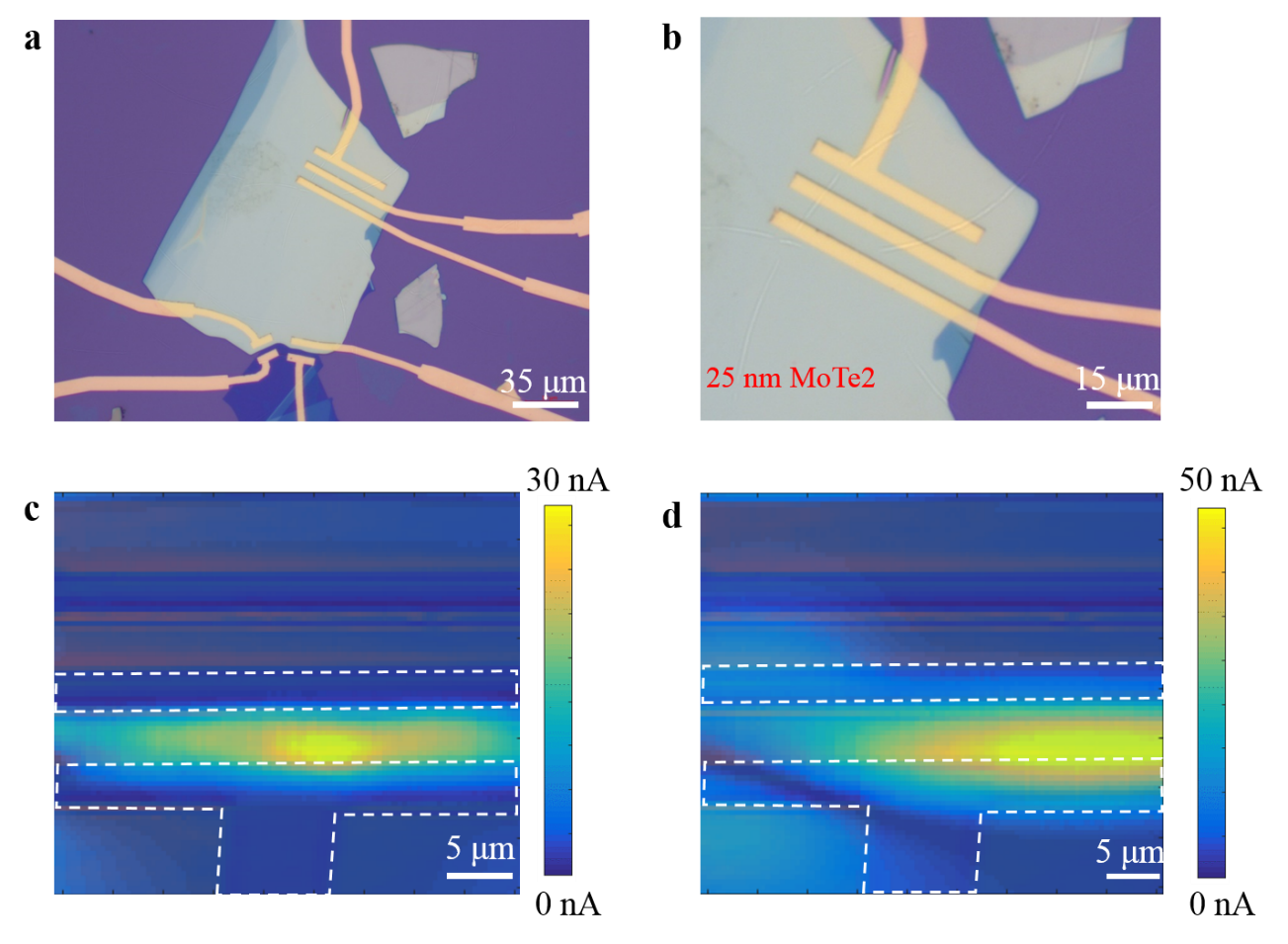


**Fig. S5** (a) (b) Low and high magnification optical images of pure 25 nm MoTe_2_ device; (c) (d) SPCM images of the MoTe_2_ photodetector excited by 488 nm CW and 780 nm pulsed laser beams.

1. **Electrical transport characteristic properties of graphene/MoTe_2_ heterojunction devices.**

I_ds_-V_ds_ output and I_ds_-V_g_ transfer characteristic performance of graphene/MoTe_2_ heterojunction devices were measured to compared with the pure MoTe_2_ device. As shown in Fig. S6 (a), in construction of heterojunction, the output curves at different gate voltages still display a linear current change with a higher current intensity than pure MoTe_2_ devices, which indicates that the semimetallic graphene serving as an electrode does not form a strong band-bending with the MoTe_2_ flake at the junction, but improves the contact between the MoTe_2_ and the Cr/Au electrode. In transfer characteristics (Fig. S6 (b)), the graphene/MoTe_2_ heterojunction device demonstrates the similar behavior of a pure MoTe_2_ transistor with a slightly reduced on-off ratio.


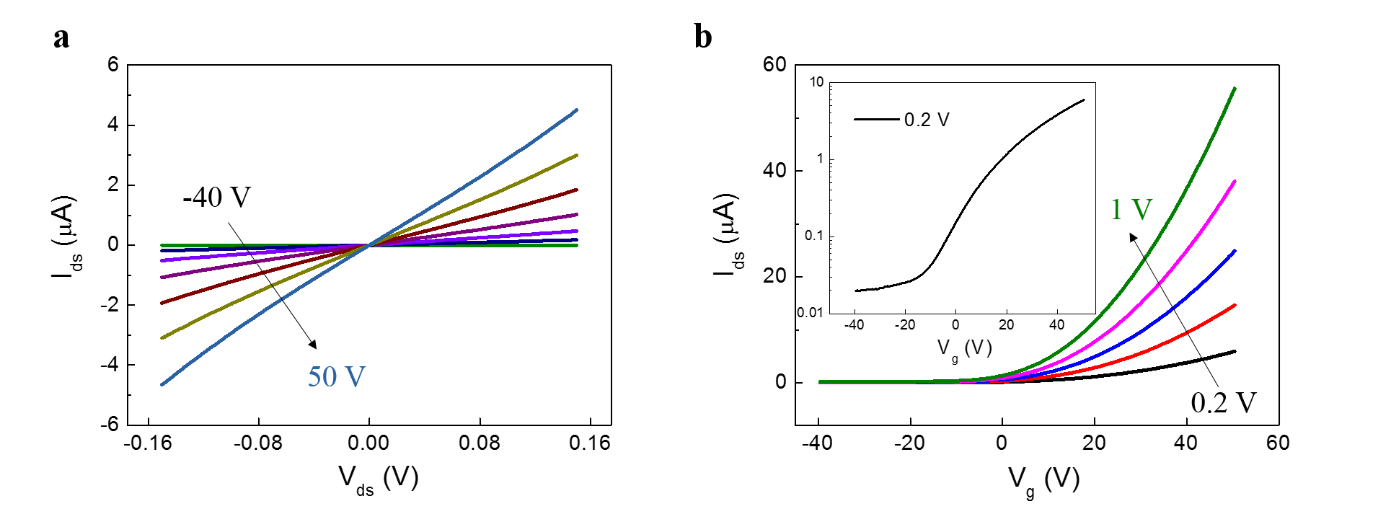


Fig. S6 (a) I_ds_-V_ds_ output characteristics of the heterojunction device at different gate voltages; (b) I_ds_-V_g_ transfer characteristics of the heterojunction device at different drain voltages. Inset: Corresponding transfer characteristics at semi-log scale. The V_ds_ is set to 0.2 V.

1. **Probe power and gate voltage dependent TRPC in graphene/ 25 nm MoTe_2_ heterojunction device.**

We normalized and fitted TRPC decay curves in probe power (Fig. S7) and gate dependent (Fig. S8) measurements, and all the curves exhibit two distinct time constants with the fast one remaining at about 15 ps.


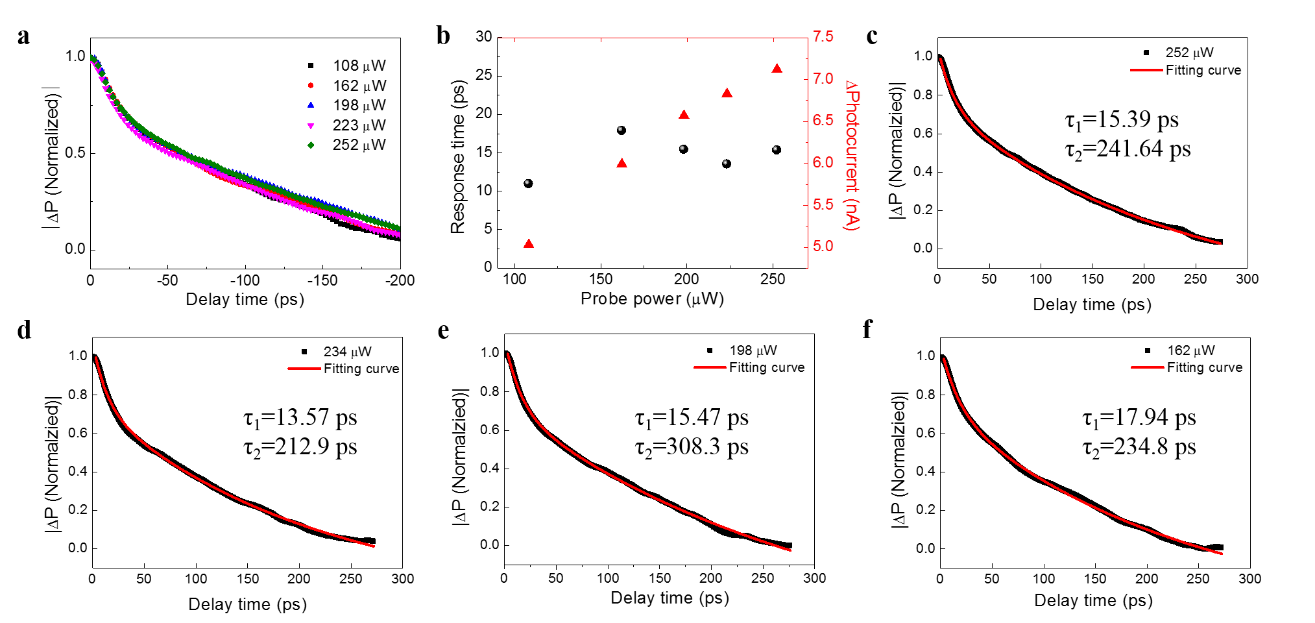


**Fig. S7** (a) Normalized TRPC decay curves in the Gr/25 nm MoTe_2_ heterojunction at probe powers from 252 μW to 108 μW; (b) Response time and ∆Photocurrent as a function of probe power; (c-f) Fitted |∆P| decay curves at different probe powers, where every TRPC decay curve exhibits two distinct time constants independent of the probe power.


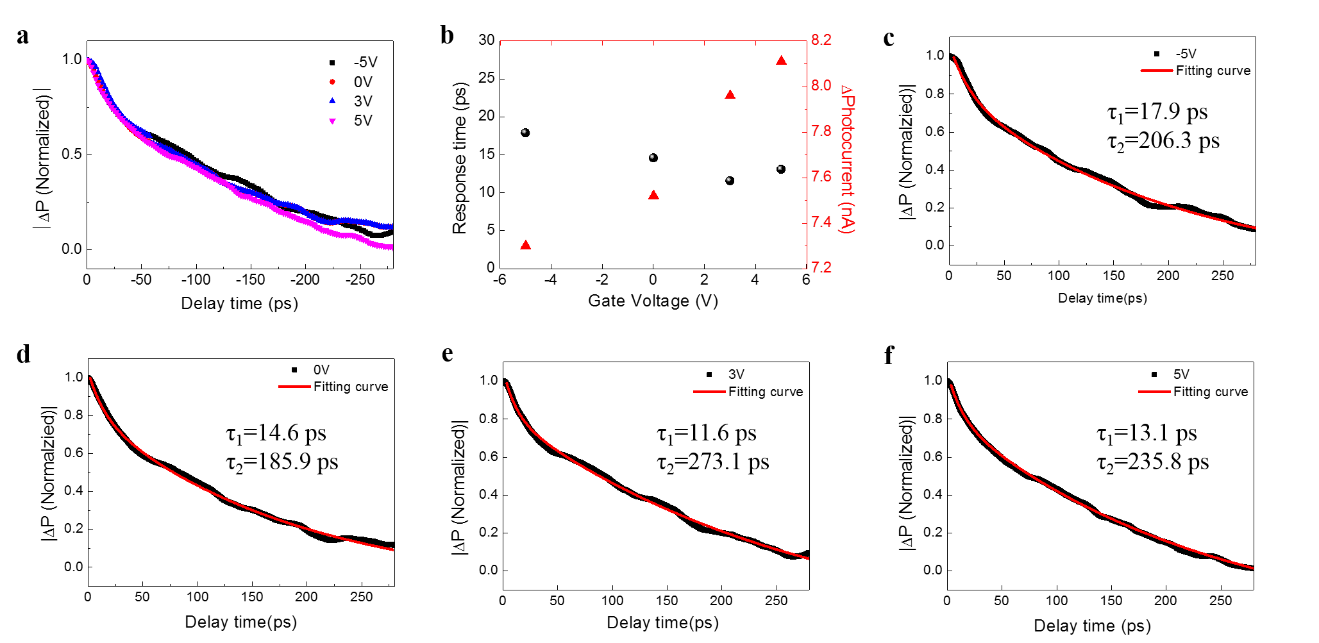


**Fig. S8** (a) Normalized TRPC decay curves in the Gr/25 nm MoTe_2_ heterojunction at gate voltages from -5 V to 5 V; (b) Response time and ∆Photocurrent as a function of gate voltage; (c-f) Fitted |∆P| decay curves at different gate voltages.

1. **Probe power dependent TRPC in graphene/ 35 nm MoTe_2_ heterojunction device.**

We also performed the TRPC measurements in the graphene/ 35 nm MoTe_2_ heterojunction photodetector. The results also exhibit two distinct time constants, where the fast one remains at 15 ps similar to the phenomenon observed in the graphene/ 25 nm MoTe_2_ heterojunction, and the slow one corresponds to the intrinsic response time of the 35 nm thick MoTe_2_ (around 1 ns).


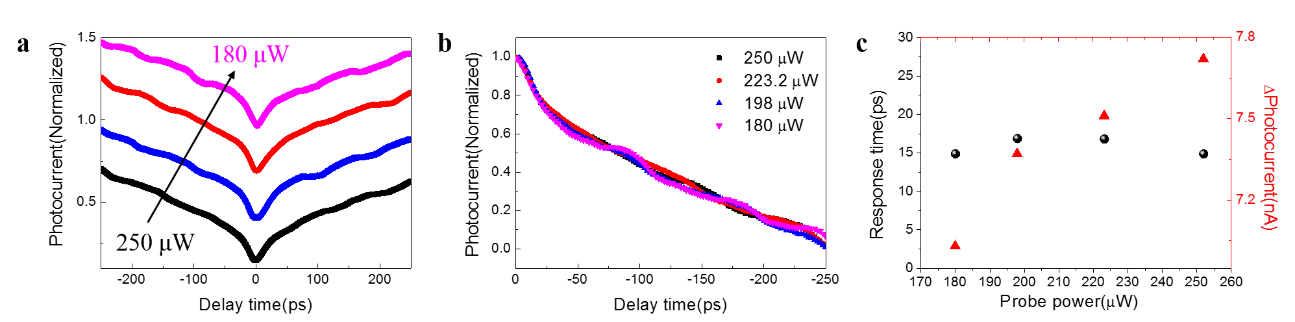


**Fig. S9** (a) TRPC in the Gr/35nm MoTe_2_ heterojunction recorded with the probe power varying from 250 μW to 180 μW; (b) Normalized photocurrent (|∆P|) decay curves at different probe powers; (c) Response time and ∆Photocurrent as a function of probe power.

1. **Probe power and gate voltage dependent TRPC in the 25 nm thick MoTe_2_ device.**


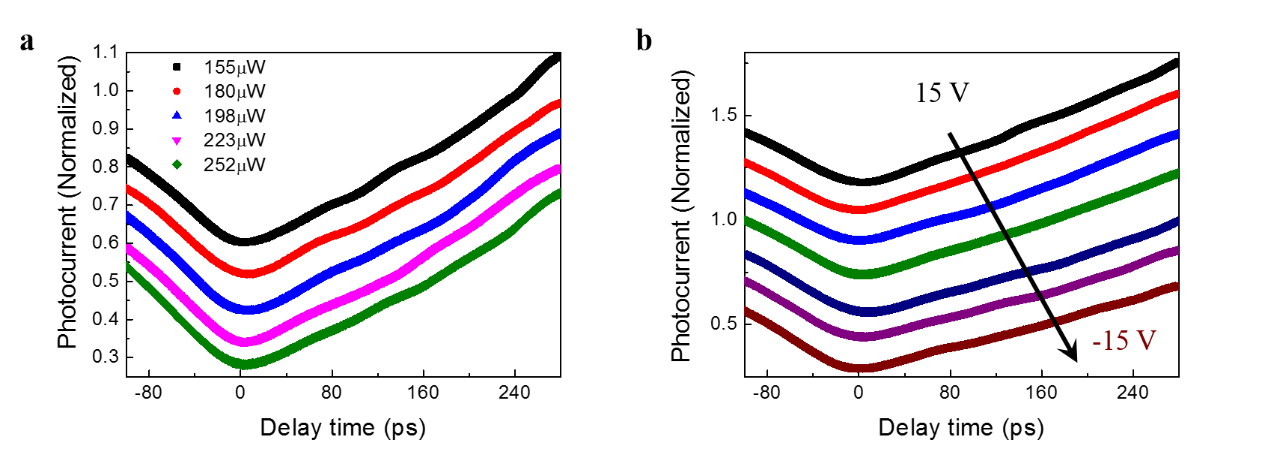


**Fig. S10** (a) TRPC measurement in the pure 25 nm thick MoTe_2_ device with the probe power varying from 252 μW to 155 μW; (b) TRPC recorded at different gate voltage changing from 15 V to -15 V. The TRPC measurements show a single decay (recover) component and it is independent of the probe power and gate voltage.

**Reference**

1. C. G. Zhu, X. X. Sun, H. W. Liu, et al., Nonvolatile MoTe_2_ p-n Diodes for Optoelectronic Logics, ACS Nano 13 (2019) 7216-7222.
